# The Relationship Between Nurses' Quality of Work-Life on Organizational Loyalty and Job Performance in Saudi Arabian Hospitals: A Cross-Sectional Study

**DOI:** 10.3389/fpubh.2022.918492

**Published:** 2022-07-12

**Authors:** Reem N Al-Dossary

**Affiliations:** Nursing Education Department, College of Nursing, Imam Abdulrahman Bin Faisal University, Dammam, Saudi Arabia

**Keywords:** leadership, nurses' job performance, organizational loyalty, quality of work-life, Saudi

## Abstract

**Purpose:**

The purpose of this study is to analyze the relationship between quality of work-life on the organizational loyalty and job performance in Saudi Arabia.

**Methods:**

This study used a cross-sectional design for collecting the data related to the nurses' quality of work-life, organizational loyalty, and job performance from nursing staff in Saudi Arabian hospitals. Three questionnaires were used in this study, which includes Quality of Work Life Scale (QWLS), Organizational Commitment Questionnaire (OCQ), and Individual Work Performance Questionnaire (IWPQ). An online version of the survey questionnaire was generated using the Google survey, to which a link is generated for collecting data. At the end of the survey, 243 responses were received. After removing the incomplete responses, 209 responses were considered for the data analysis. The statistical techniques including *t*-tests and Pearson's correlation were used in the data analysis.

**Results:**

Nurse managers reflected good quality of life, and high loyalty toward their employers, and also reflected good job performance levels. However, staff nurses reflected poor quality of work-life, organizational loyalty, and job performance. Training and development had strong positive correlation with continuance commitment (*r* = 0.628, *p* < 0.01). Job satisfaction and job security held strong positive correlation with task performance (*r* = 0.601, *p* < 0.01) and contextual performance (*r* = 0.601, *p* < 0.01).

**Conclusion:**

Quality of work-life, organization loyalty, and job performance are positively correlated, and poor quality of work-life can negatively impact job performance and organizational loyalty of nurses.

## Introduction

Saudi Arabia has been transforming its economy from an oil-based economy to knowledge-based economy through various initiatives in different sectors through vision 2030 and Saudization programs. As a result of which large structural and operational transformations are being taking place in different sectors (1, 2). One of the important approaches in this aspect is Saudization, which is aimed at empowering the Saudi nationals and increasing their participation in various industries in order to reduce the dependency on expatriates (3). Out of the 35 million population, 13.49 million are expatriate workers in Saudi Arabia (4).

Healthcare is one of the important areas of focus in vision 2030, aiming for rapid digitalization and structural transformation for achieving improved operational efficiency and sustainability. Nurses are the largest workforce in the healthcare sector, who are very important for effective functioning of healthcare as they are directly involved in taking care of the patients. Currently, there are 196,701 nurses working in various Saudi Arabian hospitals, out of which 112,317 nurses are non-Saudi, and only 42.9% of the total nursing work force includes Saudi citizens (5). Furthermore, the availability of nurses according to population needs is very low in Saudi Arabia (5.5 nurses per 1,000 population), compared to other countries like the UK (7.9 nurses per 1,000 population) and Switzerland (18 nurses per 1,000 population), but it reflects good proportion compared to its neighbors including Bahrain (2.4 nurses per 1,000 population) and the UAE (3.1 nurses per 1,000 population)0.5 (6, 7), Considering the projected nursing graduates in the next 2 years, there is a need for additional 185,722 expatriate nurses to achieve the target of having one nurse per 200 Saudi citizens (8) Given the current statistics, it is evident that there is a huge demand for nursing workforce in Saudi Arabia, and with limited local work force, the high dependency on expatriate nursing workforce still exists in the country.

Due to large expatriate workforce and limited Saudi citizens' participation, there are various challenges identified in the nursing work life and their job performance in Saudi Arabia. For instance, managing the diverse workforce can be challenging for the nurse managers. Furthermore, issues such as poor working conditions, limited opportunities, and poor image of nursing among Saudi citizens makes nursing profession least preferred among Saudis (9). In addition, due to the cultural influence, most of the Saudi women do not consider nursing as their career option (10, 11).

Focusing on the expatriate nurses, several issues such as poor living and working conditions, cultural differences, communication problems, complex social living conditions are affecting their work-life in the country (12), as a result of which their satisfaction levels and loyalty are decreasing. For instance, it was identified that many expatriate nurses initially come to Saudi Arabia even though the working and living conditions are poor, only to gain enough work experience, so that they can later move to the developed countries such as the US and the UK, where they can experience better work-life conditions (13). Therefore, there are various factors that affect both Saudi and non-Saudi nurses in relation to the quality of work-life and their satisfaction levels (14, 15).

Furthermore, sustainability, one of the main objectives of vision 2030 may not be achieved if there is a poor quality of work-life, job dissatisfaction, decreasing retention rates, poor loyalty, and poor job performance in nursing sector. As a part of Saudization, there is also a need to increase the attractiveness of nursing among the career options in Saudi Arabia. Therefore, there is an immediate need to assess various issues and the relationship between various influencing factors associated with nursing staff in Saudi Arabia.

## Literature Review

**Figure 1:**
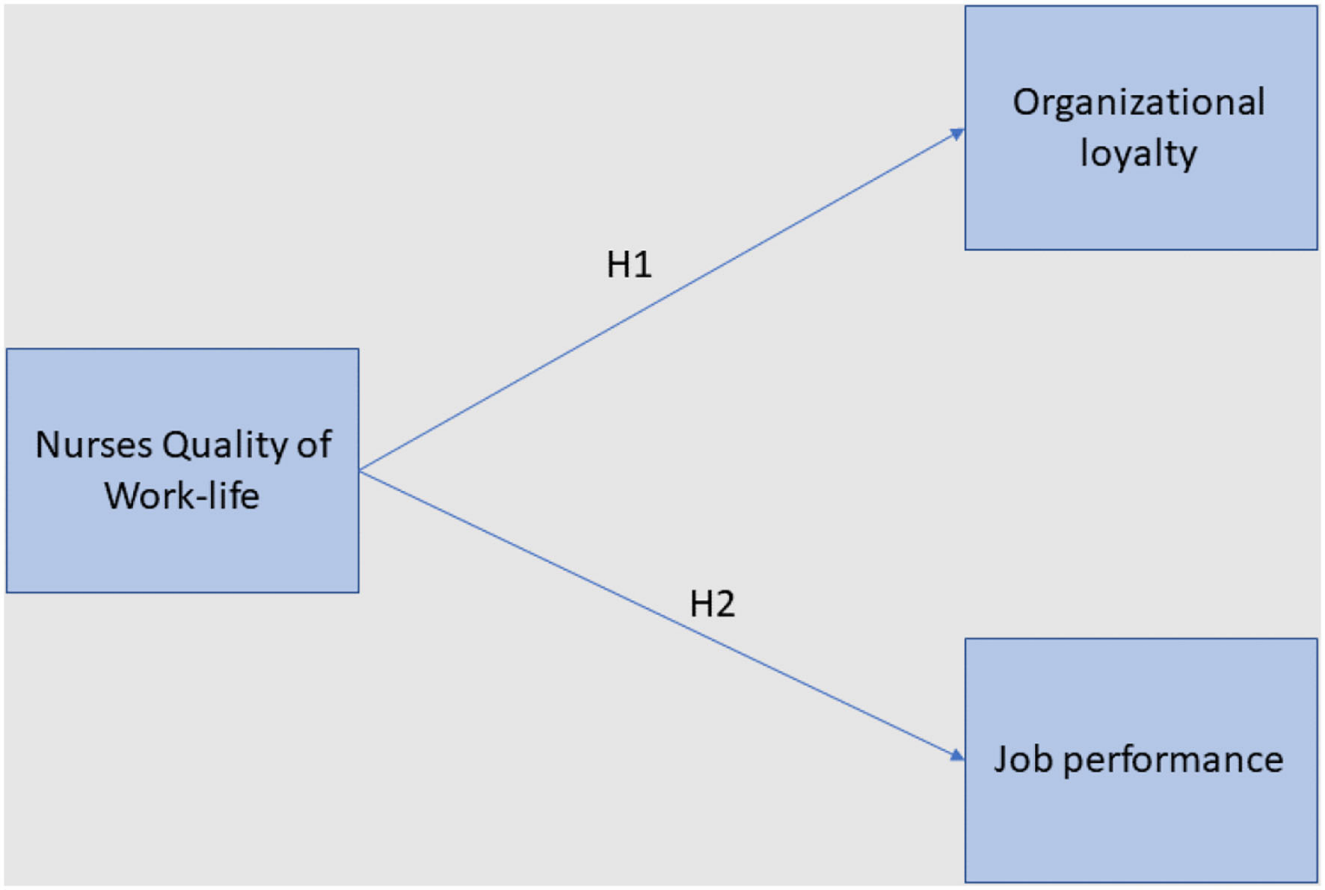
Study model.

Nurses' job satisfaction, which can be influenced by the quality of work-life was identified to be significantly correlated with commitment (16). Therefore, the poor quality of work conditions may affect the commitment or loyalty of nurses toward their organization. Furthermore, it was identified that the relationship between the nurses' quality of work-life and turnover intention was partially mediated by organization loyalty, as quality of work-life is significantly influences organizational loyalty (17). The previous studies (18, 19) have identified that work-related issues such as lack of managerial effort to improve the work environment of nurses, seeking perfectionism, support from the ward manager, salary, the relationship at work with other nurses, communication, relationship with team members, and the fairness of shift work between nurses can significantly affect the loyalty and job performance of nurses in Saudi Arabian hospitals. Communication between nurses and between nurses and patients plays an important role in improving the quality of work life, and communication issues were identified to be significantly correlated with the nurses' dissatisfaction (15). Lower satisfaction levels among the nurses may lead to decreased organization loyalty, while higher satisfaction levels may lead to increased job performance (16), whereas, improved job performance is positively correlated with organizational commitment (17). Furthermore, lower satisfaction levels may result in increased turnover intentions, indicating poor organizational loyalty (17). Moreover, the poor quality of work life such as high stress levels can adversely affect the nurses' job performance (20). Although the previous studies have identified relationships between organizational commitment and quality of work life; work stress and job performance, the relationship between the quality of work life, organizational loyalty, and job performance was not investigated. In this context, this study aims to analyze the relationship between quality of work-life on the organizational loyalty and job performance (as shown in Figure 1) in Saudi Arabia using following hypothesis.

H1: Nurses' Quality of work life is positively correlated with organizational loyalty.

H2: Nurses' Quality of work life is positively correlated with job performance.

## Materials and Methods

This study used a cross-sectional design for collecting the data related to nurses' quality of work life, organizational loyalty, and job performance from nursing staff in Saudi Arabian hospitals.

### Survey Instruments

Three questionnaires were used in this study, which include Quality of Work Life Scale (QWLS) (21), Organizational Commitment Questionnaire (OCQ) (22), and Individual Work Performance Questionnaire (IWPQ**)**0.2 (4). The quality of work life is a multi-dimensional construct, and QWLS includes nine different components which can effectively cover various aspects that may influence the nurses' quality of work life. The organizational commitment is a factor that can reflect organizational loyalty in terms of their affective, continuance, and normative commitments, which are included in OCQ, making it one of the effective and efficient instruments for assessing the commitment and loyalty factors. Similarly, IWPQ includes three contexts: task performance, contextual performance, and counterproductive performance, which can be aptly applied in the context of nursing job. Considering the relevance and applicability, the three scales such as QWLS, OCQ, and IWPQ are used for measuring quality of work life, organizational loyalty, and job performance, respectively. All the three questionnaires were embedded into a single main questionnaire. Accordingly, the main questionnaire is divided into four parts. First part focuses on the collecting participants' demographic information. Second part of the questionnaire includes 50 items from QWLS, which need to be rated on a five-point Likert scale (1: Strongly disagree; 2: Disagree; 3: Neutral; 4: Agree; 5: Strongly agree). Items one to six focus on work environment; items seven to thirteen focus on organization culture and climate; items 14–19 focus on relation and cooperation; items 20–23 focus on training and development; items 24–28 focus on compensation and rewards; items 29–33 focus on facilities; items 34–41 focus on job satisfaction and job security; items 42–47 focus on autonomy on work; and items 48–50 focus on adequacy of resources.

Third part of the questionnaire includes 18 items, measured on a 7-point Likert scale ((*SD* = 0) strongly disagree; (*MD* = 1) moderately disagree; (*LD* = 2) slightly disagree; (*O* = 3) neither disagree nor agree; (*LA* = 4) slightly agree; (*MA* = 5) moderately agree; (*SA* = 6) strongly agree) related to organizational commitment adapted from OCQ. These are further categorized into affective commitment (items 1–6), continuance commitment (items 7–12), and normative commitment (items 13–18).

Fourth part of the questionnaire includes 47 items related to job performance, measured on a 5-point Likert scale ((*N* = 0) never; (*R* = 1) rarely; (*S* = 2) sometimes; (*O* = 3) often; (*VO* = 5) very often. These are further categorized into four sections. The first section focuses on task performance and includes 13 items; second section focuses on contextual performance and includes 16 items; third section focuses on adaptive performance and includes eight items; and fourth section focuses on Counterproductive work behavior, which includes 10 items.

A pilot study was conducted with 27 nurses, and the analysis of pilot study results resulted in achieving the Cronbach's alpha of 0.83 (>0.70) indicating good internal reliability and consistency (23). An online version of the survey questionnaire was generated using the Google survey, to which a link is generated for collecting data.

### Sampling and Participants

**Figure 2:**
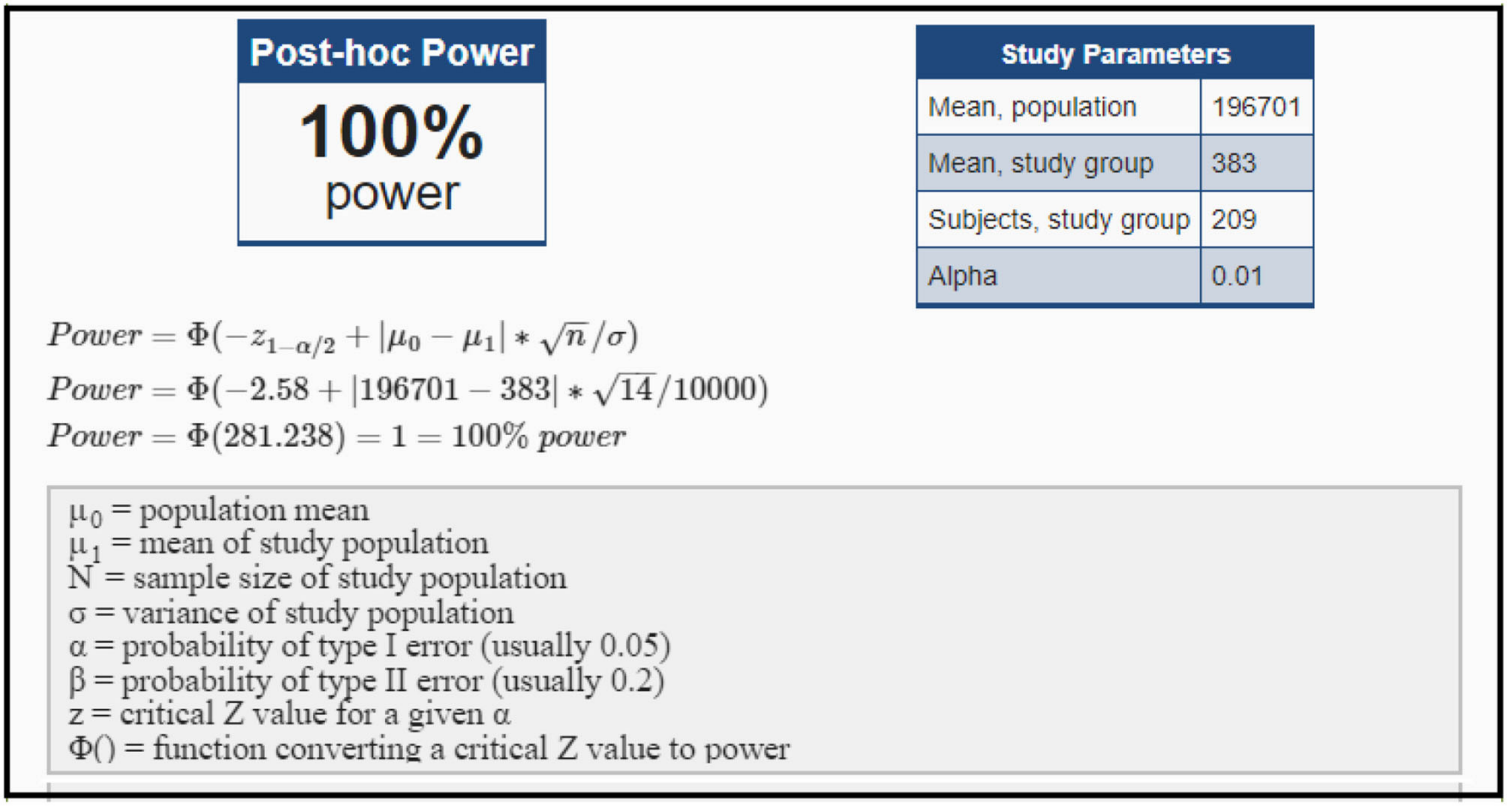
*Post-hoc* power analysis.

As the survey is targeted at nursing managers and nurses working in Saudi Arabian hospitals, the survey link is forwarded to HR administrators of 68 hospitals which include public and private hospitals. Considering 196,701 nurses working in Saudi Arabia, estimated sample was calculated using the Cochran's formula (24), at 95% *CI* and 5% of Margin of error, giving an estimated sample of 383 participants. The survey link was active from 1st December 2021 to 22nd January 2022, and at the end of the survey, 243 responses were received. After removing the incomplete responses, 209 responses were considered for the data analysis. Considering the population (196,701), with a study group of 383 nurses and considered sample of 209, the *post hoc* power analysis resulted in 100% power as shown in Figure 2.

### Data Analysis

Various statistical techniques including *t*-tests (to identify significant differences between groups), and Pearson's correlation (to identify relationships between factors) were used. Missing data were removed in order to avoid any bias in analyzing the results.

### Ethical Considerations

Participants were fully informed about the study and informed consent was taken prior to the participation. The anonymity of the participants is protected and the survey data are securely stored ensuring privacy and security. The ethical approval for the study was received from Ministry of Health, Saudi Arabia.

## Results

As shown in Table 1, the majority of the participants were women representing 74.2% of the total participants indicating higher female nursing workforce in Saudi Arabia. Among the total participants, 54.6% were expatriates and 44.4% were Saudi nurses representing higher participation of expatriates in Saudi Arabian hospitals. Furthermore, 10.1% of the participants were nurse managers and 89.9% staff nurses. The majority of the participants were young represented by 22.9% aged between 20 and 29 years, and 51.7% participants aged between 30 and 39 years. About 20.9% participants were aged between 40 and 49 years, followed by 5.3% of participants aged between 50 and 59 years. No participants were identified who were aged more than 59 years. The majority of the participants were qualified in Bachelor's degree in nursing (61.2%), followed by diploma in nursing (26.3%), master's degree in nursing (11%), and Ph.D. in nursing (1.4%). Focusing on the work experience, the majority of the participants had more than 6 years of work experience, represented by 37.8% having work experience between 6 and 10 years, and 32.5% participants having more than 10 years of work experience. In addition, 15.3% participants had 3–5 years of work experience, followed by 14.3% participants having 3 or <3 years of work experience.

**Table 1:** Participants' demographic information.

**Demographic characteristics**	***N***	**Relative frequency**
**Gender**		
Male	54	25.8%
Female	155	74.2%
**Education**		
Diploma	55	26.3%
Bachelor's degree	128	61.2%
Master's degree	23	11%
Doctorate	3	1.4%
**Age (years)**		
20–29	48	22.9%
30–39	108	51.7%
40–49	42	20.1%
50–59	11	5.3%
>59	0	0%
**Work experience**		
≤ 3 years	30	14.3%
3–5 years	32	15.3%
6–10 years	79	37.8%
>10 years	68	32.5%
**Role**		
Nurse manager	21	10.1%
Nurse	188	89.9%
**Nationality**		
Saudi	93	44.4%
Non-Saudi	116	54.6%

Analyzing the results related to the quality of work-life, the mean for all categories were identified to be between 3.0 and 3.44, indicating average-good conditions. Furthermore, differences between the nurse managers and staff nurses' perceptions of quality of work-life were presented in Table 2. The findings revealed that significant differences existed between the nurse managers and staff nurses in relation to all categories of quality of work-life, except facilities. Nurse managers perceived all categories of work-life aspects to be better compared to staff nurses, who perceived them to be average or poor.

**Table 2:** Nurse managers' and staff nurses' perceptions of quality of work life.

	**Nurse managers**		**Nurses**		***T***	***P***
	**Mean**	**SD**	**Mean**	**SD**		
Work environment	4.10	1.25	3.24	1.78	2.1,533	0.0,325^*^
Organization culture and climate	3.89	1.11	3.10	1.64	2.1,507	0.0,327^*^
Relation and cooperation	4.20	1.73	3.35	1.77	2.0,917	0.0,377^*^
Training and development	4.46	1.86	3.24	1.45	3.5,479	0.0,005^*^
Compensation and rewards	3.94	1.42	3.10	1.32	2.7,450	0.0,066^*^
Facilities	3.96	1.33	3.12	1.96	1.9,132	0.0,571
Job satisfaction and job security	4.15	1.88	3.13	1.74	2.5,274	0.0,122^*^
Autonomy of work	3.88	1.07	2.97	1.81	2.2,572	0.0,250^*^
Adequacy of resources	4.48	1.46	3.15	1.94	3.0,441	0.0,026^*^

* *Statistically significant difference*.

The overall results relating to organizational loyalty reflected poor levels, which can be analyzed from low mean ratings for affective commitment (Mean = 2.68, *SD* = 1.83), continuance commitment (Mean = 2.72, *SD* = 1.64), and normative commitment (Mean = 2.77, *SD* = 1.29). Table 3 presents the difference of opinions between staff nurses and nurse managers. The statistically significant differences were observed between nurse managers and staff nurses in all types of commitments. While nurse managers reflected greater loyalty, nurses reflected low levels of loyalty. Continuance commitment (Mean = 4.67, *SD* = 1.78) and normative commitment (Mean = 4.64, *SD* = 1.37) were high among nurse managers compared to affective commitment (Mean = 3.51, *SD* = 1.44). Among nurses all types of commitment were identified to be similar which can be analyzed from mean ratings: affective commitment (Mean = 2.59, *SD* = 1.64), continuance commitment (Mean = 2.50, *SD* = 1.93), and normative commitment (Mean = 2.56, *SD* = 1.86).

**Table 3:** Nurse managers' and staff nurses' perceptions of organizational loyalty.

	**Nurse managers**		**Nurses**		***T***	***P***
	**Mean**	**SD**	**Mean**	**SD**		
Affective commitment	3.51	1.44	2.59	1.64	2.4,656	0.0,145^*^
Continuance commitment	4.67	1.78	2.50	1.93	4.9,224	0.0,001^*^
Normative commitment	4.64	1.37	2.56	1.86	4.9,714	0.0,001^*^

* *Statistically significant difference*.

Similarly, the results related to job performance were also identified to be poor in relation to all sub-categories such as task performance (Mean = 2.3, *SD* = 1.41), contextual performance (Mean = 2.49, *SD* = 1.39), adaptive performance (Mean = 2.65, *SD* = 1.62), and counterproductive work behavior (Mean = 1.26). In similar to organizational loyalty, statistically significant differences were observed among nurse managers and staff nurses in relation to all sub-categories of job performance as shown in Table 4. Contextual (Mean = 3.63, *SD* = 1.41) and adaptive (Mean = 3.67, *SD* = 1.69) performances were rated to be high by nurse managers; while staff nurses rated all types of performances to be poor. Interestingly, counterproductive work behavior was rated to be very poor by both nurse managers and nurses although differences existed between both groups.

**Table 4:** Nurse managers' and staff nurses' perceptions of job performance.

	**Nurse managers**		**Nurses**		***t***	***P***
	**Mean**	**SD**	**Mean**	**SD**		
Task performance	2.83	1.52	2.25	1.02	2.3,374	0.0,204^*^
Contextual performance	3.63	1.41	2.36	1.19	4.5,249	0.0,001^*^
Adaptive performance	3.67	1.69	2.53	1.24	3.8,399	0.0,002^*^
Counterproductive work behavior	1.61	0.43	1.22	0.63	2.7,628	0.0,062^*^

* *Statistically significant difference*.

Table 5 presents Pearson correlation coefficients between the sub-scales of QWLS, and OCQ and IWPQ, reflecting significant correlations at 99% confidence interval. Strong positive correlations were observed between various sub-scales. Training and development had strong positive correlation with continuance commitment (*r* = 0.628, *p* < 0.01). Compensation and rewards held strong positive correlation with continuance commitment (*r* = 0.612, *p* < 0.01) and normative commitment (*r* = 0.601, *p* < 0.01). Autonomy of work held strong positive correlation with affective commitment (*r* = 0.607, *p* < 0.01) and normative commitment (*r* = 0.759, *p* < 0.01). Adequacy of resources held strong positive correlation with continuance commitment (*r* = 0.786, *p* < 0.01) and normative commitment (*r* = 0.906, *p* < 0.01). Based on the findings (Table 5), it can be observed (‘*r*' value <0.5 for most of the relations) that nurses' quality of life moderately correlated with organizational loyalty, reflecting the hypothesis (H1) to be false.

**Table 5:** Correlations between Quality of Work Life Scale (QWLS) subscales, Organizational Commitment Questionnaire (OCQ), and Individual Work Performance Questionnaire (IWPQ) subscale using the Pearson product-moment.

	**Affective commitment**	**Continuance commitment**	**Normative commitment**	**Task performance**	**Contextual performance**	**Adaptive performance**	**Counter productive work behavior**
Work environment	0.435^**^	0.583^**^	0.514^**^	0.450^**^	0.433^**^	0.396^**^	0.010^**^
Organization culture and climate	0.480^**^	0.477^**^	0.570^**^	0.446^**^	0.463^**^	0.405^**^	0.039^**^
Relation and cooperation	0.474^**^	0.590^**^	0.598^**^	0.378^**^	0.470^**^	0.348^**^	−0.009^**^
Training and development	0.446^**^	0.628^**^	0.598^**^	0.358^**^	0.446^**^	0.420^**^	0.322^**^
Compensation and rewards	0.502^**^	0.612^**^	0.601^**^	0.421^**^	0.548^**^	0.520^**^	0.238^**^
Facilities	0.463^**^	0.608^**^	0.568^**^	0.381^**^	0.511^**^	0.586^**^	0.219^**^
Job satisfaction and job security	0.517^**^	0.627^**^	0.569^**^	0.601^**^	0.606^**^	0.592^**^	0.183^**^
Autonomy of work	0.607^**^	0.573^**^	0.759^**^	0.618^**^	0.640^**^	0.715^**^	−0.002^**^
Adequacy of resources	0.449^**^	0.786^**^	0.906^**^	0.571^**^	0.753^**^	0.861^**^	−0.023^**^

** *Correlation is significant at the 0.01 level (2-tailed)*.

In relation to the relationship between quality of work-life and job performance, both positive and negative correlations were identified. The job satisfaction and job security held strong positive correlation with task performance (*r* = 0.601, *p* < 0.01) and contextual performance (*r* = 0.601, *p* < 0.01). Autonomy of work held strong positive correlation with task performance (*r* = 0.618, *p* < 0.01), contextual performance (*r* = 0.640, *p* < 0.01), and adaptive performance (*r* = 0.715, *p* < 0.01). Adequacy of resources held strong positive correlation with contextual performance (*r* = 0.753, *p* < 0.01) and adaptive performance (*r* = 0.861, *p* < 0.01). Negative correlations were identified between QWL sub-classes such as relation and cooperation, autonomy of work, adequacy of resources, and job performance sub-class including counterproductive work behavior. Based on the findings (Table 5), it can be observed (‘*r*' value <0.5 for most of the relations) that nurses' quality of life moderately correlated with job performance, reflecting the hypothesis (H2) to be false.

## Discussion

The main findings revealed that the overall quality of work-life and organizational loyalty was very poor. The job performance reflected the average performance levels of the participants. In all the aspects such as quality of work-life, organizational loyalty, and job performance, statistically significant differences were observed between the nurse managers and staff nurses. Nurse managers reflected good quality of life, and high loyalty toward their employers, and also reflected good job performance levels. However, staff nurses reflected poor quality of work-life, organizational loyalty, and job performance. As nurse managers are less involved in direct care operations because of the main focus on nursing management, it is possible that their work-life, loyalty, and performance may be different from that of staff nurses. Furthermore, differences in types of work and high salaries of nurse managers may lead to more loyalty compared to staff nurses. Similarly, high commitment levels of nurse managers were identified in a similar study (25), when compared to the commitment levels of staff nurses.

Considering the quality of work-life of nurses, several studies (26–32) have indicated poor quality of work life levels in similar with the findings in this study. Aspects identified in this study such as training and development, relationship between team members, job satisfaction, and work environment were identified to be more influencing factors related to the quality of work-life in similar to the previous study in Saudi Arabia (26). In relation to organizational loyalty, poor levels of commitment were identified in the previous studies (13, 25), which can be related with the low levels of quality of work-life analyzed from the findings in this study and also previous studies (26–32). Task performance, which can be analyzed from the performance of given basic nursing tasks in terms of quality and quantity, was identified to be reasonable among the nurse managers and nurses. Contextual performance (characterized by behaviors that support the organizational, social, and psychological environment in which the technical core must function) and adaptive performance (the extent to which an individual adapts to changes in the work role or environment) were identified to be high for managers and low for nurses. Behaviors such as demonstrating effort, facilitating peer and team performance, cooperating, and communicating, reflecting contextual performance are mostly reflected in nurse managers as they need to coordinate with all team members, and it might be the reason why nurse managers rated it to be high compared to staff nurses. Similarly, nurse managers are held with the responsibility to implement changes, as a result they need to be more adaptive so that they can enforce the changes on team members, and it may be the reason why nurse managers rated adaptive performance to be high compared to staff nurses. Counter-productive behaviors (negative behaviors that affect work/tasks/activities) were rated to be low by both nurse managers and nurses. Similar findings in relation to job performance can be observed from the previous studies (33–37), reflecting that job performance is often linked with work-life quality.

Accordingly, counterproductive behavior is negatively correlated with different work-life subscales such as relations and cooperation, autonomy of work, and resources adequacy, and reflected a weak positive correlation with all other sub-scales of work-life subscales. However, the significant positive correlations were established between quality of work life and job performance, such as task, contextual, and adaptive performance. Job satisfaction, autonomy of work, and resource adequacy reflected more strong correlations with task, contextual, and adaptive performance; and all types of commitment. As identified in the previous studies (12, 14, 15, 26, 38), job satisfaction, one of the important ‘quality of work-life' subscale was identified to be significantly influencing job performance and organization loyalty. Thus, it can be analyzed and concluded that quality of work-life can moderately influence the organization loyalty and job performance of the nurses in Saudi Arabia, based on the hypothesis analysis.

Overall, this study has both theoretical and practical implications. Theoretically, it contributes to the literature, addressing the research gaps in nursing research in Saudi Arabia. Although the factors, organizational loyalty, job performance, and quality of work-life of nurses were addressed in different research separately, no efforts were taken in linking these factors and assessing the relationship between them (39, 40). This study addresses the above issue. Secondly, it has practical implications as it provides valuable findings in relation to quality of work life, organization loyalty, and commitment, which can help decision-makers in improving the nurses' work-life (as the results revealed average quality of work life, poor commitment, and job performance, especially among nurses but not nurse managers) and their performance by taking necessary measures such as deploying transformational leadership styles, providing support and training, increasing pay, rewards, etc., to attract more committed nursing employees as an approach toward achieving vision 2030 objectives.

However, there are few limitations in this study. Firstly, the sample identified in this study is lower than the estimated sample; therefore, results should be generalized with care. Secondly, as there was no prior study identified investigating the three factors: quality of work-life organizational loyalty, and job performances of nurses, the discussion is limited with comparison with studies focusing either one or two considered factors. Thirdly, the study is limited to the nursing staff in Saudi Arabia; therefore, the generalization of results, especially in other similar countries should be done with care. These limitations can be addressed in future studies. Future studies can focus on analyzing the relationship between these three factors in other countries, and may include more diverse and higher sample population in order to generalize the results, and address the issue of limited research in this area. In addition, multiple relationships between other factors such as satisfaction, turnover intentions, commitment, and attitudes may be included in future studies.

## Conclusion

The purpose of this study is to analyze the relationship between quality of work-life on the organizational loyalty and job performance in Saudi Arabia, thereby addressing the research gaps identified in this area. Findings have revealed that Quality of work-life is moderately correlated with organization loyalty and job performance. Poor quality of work-life can negatively impact job performance and organizational loyalty of nurses. Considering the fast-changing work-life of nurses being influenced by various factors, it may be assumed that they may be under stress, which may significantly affect their work-life. This in turn can influence organizational loyalty and job performance, affecting the overall healthcare services. Therefore, it is important that the measures need to be developed for addressing the issues in nurses' quality of work-life, thereby improving the job performance and loyalty. However, further studies are needed to establish clear a relationship between these factors in various contexts.

## Data Availability Statement

The raw data supporting the conclusions of this article will be made available by the authors, without undue reservation.

## Ethics Statement

The studies involving human participants were reviewed and approved by the Ministry of Health.

## Author Contributions

The author confirms being the sole contributor of this work and has approved it for publication.

## Conflict of Interest

The author declares that the research was conducted in the absence of any commercial or financial relationships that could be construed as a potential conflict of interest.

## Publisher's Note

All claims expressed in this article are solely those of the authors and do not necessarily represent those of their affiliated organizations, or those of the publisher, the editors and the reviewers. Any product that may be evaluated in this article, or claim that may be made by its manufacturer, is not guaranteed or endorsed by the publisher.
